# Emerging concepts for PI3K/mTOR inhibition as a potential treatment for osteosarcoma

**DOI:** 10.12688/f1000research.8228.1

**Published:** 2016-07-06

**Authors:** Michael W. Bishop, Katherine A. Janeway

**Affiliations:** 1 Department of Oncology, St Jude Children's Research Hospital, Memphis, TN, USA; 2 Department of Pediatrics, University of Tennessee Health Science Center, Memphis, TN, USA; 3 Boston Children's Cancer and Blood Disorders Center, Boston, MA, USA

**Keywords:** osteosarcoma, PI3K, mTOR, inhibition treatment

## Abstract

Patients with metastatic and recurrent osteosarcoma fare poorly, and new therapeutic strategies are needed to improve survival. Several recent complementary genomic and pathway analyses of both murine and human osteosarcoma have revealed common aberrations of the phosphoinositide 3-kinase (PI3K)/mammalian target of rapamycin (mTOR) pathway in osteosarcoma. Preclinical data demonstrate that inhibition of PI3K and mTOR with either a combination of single agents or dual inhibiting compounds can decrease cell proliferation and induce cell cycle arrest and apoptosis. With a lack of available clinical agents active in osteosarcoma, PI3K/mTOR inhibition represents a potential vulnerability in osteosarcoma that warrants clinical investigation.

## Introduction

Osteosarcoma is the most common malignancy of bone diagnosed in children and adolescents, with an age-adjusted incidence of 4.4 new cases per million each year ^1^. Contemporary studies estimate that with the use of surgery and multimodal chemotherapy with high-dose methotrexate, cisplatin, and doxorubicin, 65 to 70% of patients are able to achieve long-term cure ^2^. However, patients who present with metastatic disease fare poorly, with survival rates less than 30% ^3– 5^. Furthermore, for patients who relapse, survival is less than 20%, and cure is nearly impossible if surgical complete remission cannot be achieved ^6, 7^. Several avenues for augmenting treatment are currently under preclinical or clinical investigations, supported by recent biologic and genomic data. Among these, interest has been raised for the use of drugs targeting the phosphoinositide 3-kinase (PI3K)/mammalian target of rapamycin (mTOR) pathway as a potential vulnerability. Aberrations of this pathway have previously been described in osteosarcoma, such as *PTEN* deletion ^8^ and *PIK3CA* mutations ^9^, but were observed at relatively low frequency. However, contemporary biologic studies now reveal more frequent alterations of this pathway. The following brief review will highlight recent analyses supporting the role of PI3K/mTOR inhibition as an area ripe for further exploration in osteosarcoma.

## Genetic studies reveal potential role for PI3Km/TOR inhibition in osteosarcoma

As part of efforts to identify targets for novel therapeutic agents, institutional and collaborative group efforts to characterize the genomic landscape of osteosarcoma have been conducted with the hope of identifying targetable recurrent aberrations. Numerous genomic and epigenetic analyses have revealed the striking genomic complexity and heterogeneity among osteosarcoma samples but have also elucidated a few common themes including alterations of *TP53* and/or *RB1* in most samples, and distinct chromosomal regions of hypermutation (“kataegis”) ^10, 11^. However, a recent complementary genomic and pathway analysis identified PI3K/mTOR pathway aberrations in a subset of osteosarcoma samples. Heuristic analysis of whole genome, exome, and RNA sequencing data from 59 osteosarcoma tumors revealed alterations in the PI3K/mTOR pathway in 24% of samples that included aberrations of *PTEN*, *TSC2, PIK3R1, PIK3CA*, and several other genes. Using a comparative oncology approach, whole exome sequencing of a *Tp53/Rb1* conditionally deleted osteosarcoma mouse model, somatic mutations in *PTEN* and *PIK3R1* were observed in both murine and human tumors. Furthermore, a genome-wide shRNA screen of a primary murine osteosarcoma cell line identified 172 enriched genes including *Pik3ca* and *Mtor*, with inhibition of murine osteosarcoma cells ^11^. Based on this information, two dual PI3K/mTOR inhibitors (GSK2126458, BEZ-235) and a PIK3CA-specific inhibitor (PIK75) were tested against human and murine-derived cell lines. All three drugs inhibited cell proliferation in all cell lines; PIK75 and GSK2126458 induced apoptosis as demonstrated by caspase 3/7 activation and poly(ADP-ribose) polymerase (PARP) cleavage.

In a separate systematic analysis, whole-genome siRNA screening of primary osteosarcoma cell cultures derived from a genetically engineered murine model revealed enrichment in pathways associated with protein translation and mTOR signaling. A small molecule/kinase inhibitor screen of murine-derived osteosarcoma cell lines revealed activity in compounds (PIK-75, GSK2126458, and BEZ-235) targeting PI3K and mTOR and/or DNA-PK. Activity of dual PI3K/mTOR inhibitors was subsequently observed in cell death assays of cultures from primary human xenograft-derived osteosarcoma (GSK2126458, PKI-587, BEZ-235, and BGT-226). Administration of the compounds GSK2126458 and PKI-587 inhibited phosphorylation of downstream targets in a dose-dependent manner, increased the number of cells in the G _0_-G _1_ phase, and induced apoptosis in both murine and human cell lines. Combinations of PI3K- or mTOR-specific inhibitors were also evaluated, and while individual activity was not observed, combination of the PIK3CA-specific inhibitor BYL719 and everolimus yielded a synergistic interaction ^12^.

Further supporting these comprehensive analyses, the use of novel genetic screening technologies provides additional evidence for the importance of the PI3K/mTOR pathway in osteosarcoma. A *Sleeping Beauty* (SB) transposon-based forward genetic screen was performed in mice with and without somatic loss of *Trp53* to identify common insertion sites associated with the development of osteosarcoma. *Pten* was one of the most commonly mutated genes in both Trp53-SB-mutated and non-Trp53-SB-mutated tumors. *Nf2* and *Nf1*, both of which serve regulatory functions for downstream mTOR signaling, were also frequently mutated in the SB-mutated tumors. Pathway analysis identified enrichment for candidate genes in the PI3K/AKT/mTOR pathway, as well as overlap with the ErbB and ERK/MAPK pathways. Furthermore, conditional knockdown of both *Trp53* and *Pten* in a mouse model accelerated the development of osteosarcoma, and knockout of *PTEN* in an immortalized osteoblast cell line with inhibited *TP53* function led to significantly increased colony formation, suggesting that *PTEN* loss is cooperative with *TP53* dysfunction to drive osteosarcomagenesis and proliferation ^13^.

Several clinical reports describing activity of agents targeting mTORC1/2 have included patients with osteosarcoma. A recent report from the French Sarcoma Group of off-label use of targeted therapies for osteosarcoma found that those who received rapamycin (with or without cyclophosphamide) compared to a group of tyrosine kinase inhibitors (sunitinib, sorafenib, and pazopanib) had a superior progression-free survival (PFS) (hazard ratio [HR] 2.7, 95% confidence interval [CI] 1.05–7.1), although the difference in median PFS was modest (3 months vs. 1.8 months) ^14^. A phase II study of the mTOR inhibitor ridaforolimus included two osteosarcoma patients with a confirmed partial response and one patient with an unconfirmed partial response ^15^. Fifty osteosarcoma patients were enrolled on a subsequent phase III study using ridaforolimus as a maintenance therapy but were included in a cohort of bone tumors and were not analyzed separately. In a subgroup analysis, the use of ridaforolimus trended toward improved PFS for bone tumors but did not achieve statistical significance (HR 0.70, 95% CI upper limit >1); the study was not powered for subgroup analyses ^16^.

Everolimus has also demonstrated some activity in osteosarcoma; in a pediatric phase I study, one of two enrolled osteosarcoma patients experienced prolonged stable disease for eight courses ^17^. Everolimus has been shown to decrease drug-induced resistance to sorafenib via abrogation of the upregulation of mTORC1 and mTORC2 in murine models ^18^; the combination of sorafenib and everolimus in an adult phase II study in patients with recurrent osteosarcoma with measurable disease yielded a 6-month PFS of 45% ^19^. These results are in stark contrast to those observed in an historical cohort of osteosarcoma patients enrolled in phase II trials (Lagmay J *et al*., J Clin Oncol, in press). A recently completed phase I study of everolimus in combination with pazopanib for the treatment of adults with advanced solid tumors demonstrated tolerability; prolonged stable disease or partial response was observed for several patients with PI3K/AKT/mTOR pathway alterations ^20^. Not all published studies have supported activity of mTOR inhibition in osteosarcoma; prior combinations of rapamycin with cyclophosphamide and temsirolimus with the insulin-like growth factor-1 receptor (IGF-1R) antibody cixutumumab failed to demonstrate efficacy in phase II studies including recurrent osteosarcoma patients ^21– 23^. However, when viewed as a whole, the available clinical data suggest potential activity of inhibition of the PI3K/mTOR pathway in osteosarcoma, which warrants further investigation.

## Conclusions

The above data present compelling evidence of the role for dysregulation of the PI3K/mTOR pathway in osteosarcoma and suggest an opportunity for focused therapeutic strategies. Prior studies of mTOR inhibitors in osteosarcoma have demonstrated a hint of activity for targeting this pathway but were not adequate to assess the activity of PI3K/mTOR inhibition in osteosarcoma due to study design, lack of molecular correlation, and/or the number of patients enrolled. Furthermore, evidence that combinations of PI3K and mTOR inhibition can overcome patterns of resistance observed in single agent exposures and combination therapy shows potential promise for clinical activity. Given the paucity of active agents available for the treatment of recurrent and refractory osteosarcoma, inhibition of PI3K and mTOR may present a viable treatment strategy deserving of clinical investigation. It remains unclear whether the presence of aberrations within the pathway truly function as biomarkers of susceptibility to targeted agents; therefore, as a component of any future prospective studies of PI3K/mTOR inhibition, genomic analysis and assessment of activity of the pathway should be included as correlative biology studies.
